# Assessing the strength of innovations in the treatment of depression

**DOI:** 10.1192/bjp.2025.98

**Published:** 2025-11

**Authors:** Pim Cuijpers, Mathias Harrer, Toshi Furukawa

**Affiliations:** Department of Clinical, Neuro and Developmental Psychology, Amsterdam Public Health Research Institute, Vrije Universiteit Amsterdam, Amsterdam, Netherlands; Babeș-Bolyai University, International Institute for Psychotherapy, Cluj-Napoca, Romania; Department of Psychiatry and Psychotherapy, School of Medicine and Health, Technical University Munich, Munich, Germany; Kyoto University Office of Institutional Advancement and Communications, Kyoto, Japan

**Keywords:** Depression, major depressive disorder, innovation, treatment, psychotherapy and pharmacotherapy

## Abstract

Although treatments for depression are effective, many patients do not respond. Many new innovations are currently being developed, claiming to substantially improve outcomes. We propose a new method to assess the strength of these innovations. Based on response rates of current treatments, we can estimate how many treatments are needed in total to realise response in >99% of patients if they were to be offered another treatment when the previous one did not work. Using a basic model as a benchmark, we can show that none of the current innovations likely represents a ’silver bullet’ that will dramatically change the outcomes. Improvement of mental healthcare for depression needs to be done by multiple, incremental innovations. Only together can these innovations substantially improve outcomes.

Three-hundred million people worldwide suffer from depressive disorders, and although evidence-based treatments are available, current treatments can only reduce the disease burden of depression by 33%.^1^ Innovative treatments are, therefore, desperately needed. Researchers all over the world are examining many kinds of innovations, including personalised treatments, add-on digital interventions, cognitive remediation, new treatments such as esketamine and psychedelics for treatment-resistant depression, new neuromodulation approaches, better matching patients with therapists, systematic feedback from patients to therapists and many more. We all hope that one of these innovations is a ‘silver bullet’ that will dramatically increase the outcomes of treatments. In this article we will present a simple method that could help with assessing the strength of innovations.

## A benchmark for assessing the strength of innovations

Hundreds of randomised controlled trials and dozens of meta-analyses have shown that psychological and pharmacological treatments are effective in the treatment of depression.^2,3^ That does not mean, however, that every patient responds, let alone remits, after the first treatment. We found, for example, that only 42% of patients respond (defined as 50% symptom reduction) to psychological treatments for depression.^4^ Comparable outcomes have been found for pharmacological treatments. This means that many patients, maybe even more than half, are not substantially improved after their first treatment.

Patients who do not respond to the first treatment can be provided with another treatment and, when that does not work, a third treatment, a fourth treatment, etc. We can then take a group of 100 patients and aim to treat them all until they have responded. This process can be viewed as a type of absorbing Markov chain,^5^ and it allows one to calculate the total number of treatments that should be provided to this group to make them better. We could assume that the first 42% of these patients respond to the first treatment, another 42% of the remaining 58% to the second treatment, etc. To realise response in >99% of all patients, we would need to provide up to nine treatment cycles, resulting in a total of 237 treatments. Eighty percent of patients will have responded after three treatments, but the remaining patients will need up to nine treatments. This could be a realistic scenario if there were one specific treatment that would be effective for one specific patient. If the first treatment does not work for this patient, the next one could be the effective one, and otherwise the third or fourth.

**Fig. 1:**
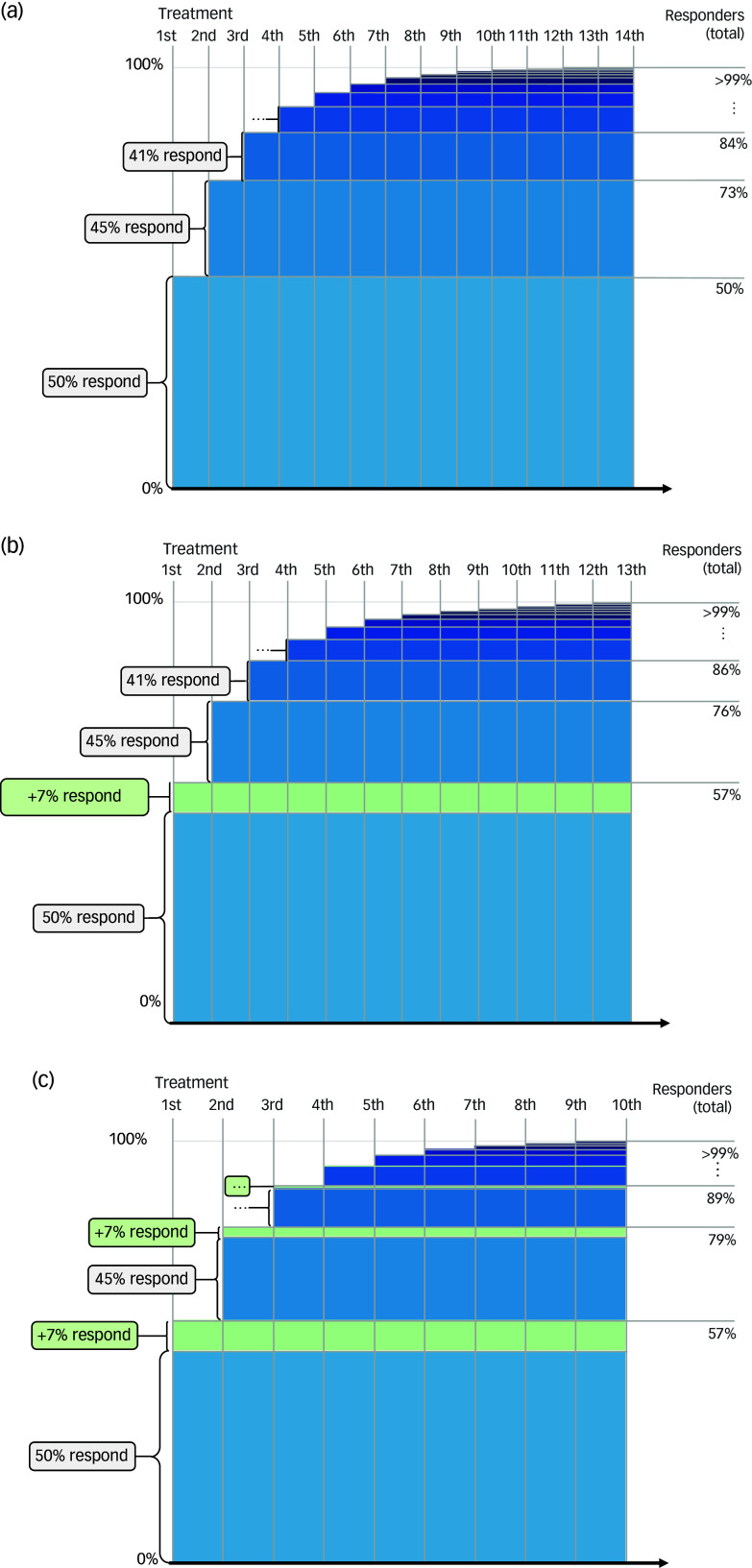
Cumulative response rates in depression treatments: three scenarios. (a) Realistic scenario: 50% response in first treatment, 10% decline for next treatments. (b) Innovation with first treatment 50% more effective (57% response). (c) Innovation with all treatments 50% more effective.

However, that is probably not the most realistic scenario, as it assumes that any new treatment is as effective as the previous one. Evidence shows that the first ever treatment is more effective than following treatments.^6^ Although there is not enough evidence to make accurate estimates about such scenarios, we can make some reasonable assumptions. We could assume that the response rate in patients seeking treatment for the first time is not 42%, but 50%, and that each new treatment is, for example, 10% less effective than the previous one. If we were to then again calculate the total number of treatments that would be needed to realise response in (>99% of) 100 patients, we would need up to 14 steps and 230 treatments in total. In this scenario, 84% of the patients have responded after three treatments, the remaining patients need up to 14 treatments and the average number of treatments per patient is 2.7. In Fig. 1(a), we have presented the number of treatments needed and the cumulative response rate for this scenario.

We could use this number of 230 treatments as a benchmark for the potential of innovations and explore how many treatments can be reduced by each innovation. Because we would need to treat the 100 patients anyway with one treatment, we can better use the number of ‘excess’ treatments, 130 (230 minus 100), as a benchmark.

## The strength of innovations

Suppose we could develop a new treatment that is substantially more effective than current treatments, for example with a standardised mean difference (SMD) of 0.45 when compared to placebo. That is 50% more than the difference between antidepressants and placebo (which has an SMD of 0.30).^2^ This would increase the response rate from 50% for the first treatment to 57%,^7^ but would not increase the effects of the second, third and later treatments. In this scenario (Fig. 1(b)), the new treatment would reduce the number of excess treatments from 130 to 110, a reduction of 15%. Even if we could double the effects of the first treatment (twice the difference between antidepressants and placebo), we would still only be able to reduce the number of excess treatments with 35% (from 130 to 85).

It would be even better if we could improve not only the first treatment, but all treatments that are offered to patients. In this scenario all treatments in each step are 7% more effective, but still decline with an increasing number of treatments (Fig. 1(b)). This could be the case, for example, for stratification of patients based on machine learning techniques, or progress feedback during psychological treatments for mental health conditions. If such an innovation could realise this improvement, the number of excess treatments would be reduced by 28% (from 130 to 93). In the unlikely case that we could double the effects of all treatments in each step, the number of excess treatments would still only be reduced with 50% (from 130 to 65).

New treatments for treatment-resistant depression of patients not responding to the first two treatments are clinically extremely useful, but the reduction in excess treatments is modest. In our benchmark scenario, 27% of patients have not responded to the first two treatments. If a random new treatment were to be offered to one of these patients, the expected response rate is 41% (the original response of 0.5 with two steps of a reduction with 10%). If we would be able to increase the effects of a treatment of this group to the effect that can be realised in people who get their first treatment (50% response), that would increase the response rate in this group only modestly (from 41 to 50%). We can also see that in the overall outcomes for this scenario there is a reduction of excess treatments of only 7% (from 130 to 121).

## Limitations

This approach can be considered mechanistic and artificial, because this is not how clinical practice works in reality. Few patients will be motivated to receive ten or more treatments before getting to the one that works for them. Clinicians in routine care are also often working beyond the limits of the existing knowledge and are trying to choose treatments or sequences of treatments based on individual characteristics. Our approach also assumes that all treatments in each of the steps have the same ‘weight’, which is certainly not true (electroconvulsive therapy (ECT) will not be acceptable to any patient as the first treatment, but it is in many severe cases after three failed treatments). Another limitation is that in our scenarios we used response as an outcome, which may not be the best indicator of clinical benefit. Although there is consensus in the field of depression that response is an important indicator of outcome,^8^ it is very well possible that a smaller reduction of symptoms is highly relevant for an individual patient. This approach also assumes that there is a linear association between the number of treatments and the decline of effectiveness, but this could just as well be exponential or completely idiographic after the first few treatments. Furthermore, these models assume that there is no relapse, while it is known that relapse rates after successful treatment are considerable in depression. Finally, all scenarios that we described have the goal to realise response in 99% of patients. That may be a aspiration to aim for, but may not be realistic. A goal of realising response in 70 or 80% of patients would also already be important from a clinical perspective.

The exact numbers needed to make exact estimates for such a model are not known, so we do not know what this model looks like in reality. The numbers also depend on the definition of response, the response rate in those receiving treatment for the first time and the reduced effectiveness of following treatments. Most of these numbers are not known. However, in the supplement, several other scenarios with varying assumptions are presented. The general conclusions about the strength of different innovations remains the same, whatever the exact numbers are.

## What innovations should we focus on?

Despite such limitations, we can draw several important conclusions from this exercise, regardless of the exact outcome of each of the scenarios. The main conclusion is that none of the current innovations likely represents a ’silver bullet’. In our main scenario a new treatment that is 50% more effective than current treatments would reduce the number of excess treatments by only 15%. An innovation that would make all treatments 50% more effective would reduce the number of excess treatments by less than 30%. These numbers may vary depending on the scenario used and the exact values used in the scenarios, but in none of the scenarios can we expect a ‘silver bullet’ that dramatically changes the outcomes.

A second major conclusion from these scenarios is that all innovations are very much needed and that no single innovation will be enough to bring the number of excess treatments close to zero. Mental healthcare can only be improved by multiple, incremental innovations. All innovations that are currently being developed can only contribute to a limited extent. Only together can such innovations substantially improve the outcomes of treatments.

## Supporting information

Cuijpers et al. supplementary material 1 Cuijpers et al. supplementary material

Cuijpers et al. supplementary material 2 Cuijpers et al. supplementary material

## Data Availability

The syntaxes and data are available in the Supplemental Material.

## Supplementary material

The supplementary material can be found at https://doi.org/10.1192/bjp.2025.98
