# Supplementary material for: Assessing the strength of innovations in the treatment of depression
Source: Br J Psychiatry. 2025 May 21;227(5):810–3. doi: 10.1192/bjp.2025.98 (PMC12550659; doi:10.1192/bjp.2025.98)
Supplement: Cuijpers et al. supplementary material 2 — Cuijpers et al. supplementary material [file S0007125025000984sup002.docx]

### Supplemental materials

In the “simple” scenario, the repeated treatment cycles can be modeled as a simple Markov chain with absorbing state $S_{0}$ (patient responds), as well as transition probabilities $p_{k}=P\left( S_{0} | S_{k} \right)$ and $1-p_{k}=P(S_{k+1}|S_{k})$. Here, $k$ is the current treatment cycle, and $p_{k}$ is the probability of responding to the $k$th treatment, which is held constant across all treatment cycles.

In this scenario, the formula to calculate the cumulative response $C_{n}$ after $n$ treatment cycles reduces to:

| $C_{n}=1-\left( 1-p \right)^{n}$ | (1) |
| --- | --- |

The number of “excess” treatment $N^{E}$ can be obtained using this formula:

| $N^{E}\approx\frac{100\times(1-\left( 1-p \right)^{n})}{p}-100$ | (2) |
| --- | --- |

If we additionally consider “decay” of the treatment response (e.g., response rates decrease by 10% with each additional treatment attempt), we obtain the following formula for the cumulative response:

| $C_{n}= \sum_{i=1}^{n} \left( \prod_{j=1}^{i-1} \left( 1-p\left( 1-d \right)^{j-1} \right) \right)p\left( 1-d \right)^{i-1}$ | (3) |
| --- | --- |

where $C_{n}$ is the cumulative treatment response, $p$ is the response rate of treatments, $d$ encodes the proportional “decay” with each treatment cycle, and $n$ is the total number of treatment cycles.

Reference:

Blitzstein, J.K., & Hwang, J. (2019). Introduction to Probability, Second Edition (2nd ed.). Chapman and Hall/CRC. https://doi.org/10.1201/9780429428357

Overview of the scenarios

|  | *Cycles* | *Total Txs* | *Excess Txs* | *Mean Txs* |  | *Reduction excess Txs* |
| --- | --- | --- | --- | --- | --- | --- |
| A. MAIN SCENARIOS |  |  |  |  |  |  |
| A.1. Simple scenario (42% response, no decline) | 9 | 237 | 137 | 2.31 |  |  |
| A.2. Realistic scenario (50% response, 10% decline) | 14 | 230 | 130 | 2.67 |  | Benchmark |
| A.3. Higher response 1^st^ treatment (57%, 10% decline) | 13 | 210 | 110 | 2.48 |  | 15% (20/130) |
| A.4. Higher response in all treatments (7%; 10% decline) | 10 | 193 | 93 | 2.11 |  | 28% (37/130) |
| A.5. Higher response 3^rd^ treatment (50%) | 13 | 221 | 121 | 2.48 |  | 7% (9/130) |
| A.6. Double effect size for first treatment (64%; 10% decline) | 11 | 185 | 85 | 2.17 |  | 35% (45/130) |
| A.7. Double response in all treatments (14%; 10% decline) | 7 | 165 | 65 | 1.72 |  | 50% (65/130) |
| B. SENSITIVITY ANALYSES (response to 1st treatment is 60%) |  |  |  |  |  |  |
| B.1. Realistic scenario (60% response, 10% decline) | 9 | 179 | 79 | 1.92 |  | Benchmark |
| B.2. Higher response 1^st^ treatment (67%, 10% decline) | 8 | 166 | 66 | 1.83 |  | 16% (13/79) |
| B.3. Higher response in all treatments (7%; 10% decline) | 7 | 158 | 58 | 1.66 |  | 27% (21/79) |
| B.4. Higher response 3^rd^ treatment (60%) | 8 | 175 | 75 | 1.84 |  | 5% (4/79) |
| C. SENSITIVITY ANALYSES (decline is 5%) |  |  |  |  |  |  |
| C.1. Realistic scenario (50% response, 5% decline) | 9 | 209 | 109 | 2.15 |  | Benchmark |
| C.2. Higher response 1^st^ treatment (57%, 5% decline) | 9 | 195 | 95 | 2.09 |  | 13% (14/109) |
| C.3. Higher response in all treatments (7%; 5% decline) | 7 | 182 | 82 | 1.85 |  | 25% (27/109) |
| C.4. Higher response 3^rd^ treatment (50%) | 9 | 206 | 106 | 2.11 |  | 3% (3/109) |
|  |  |  |  |  |  |  |

Abbreviations: Txs: treatments.
